# *N*-(2,6-Dimethyl­phen­yl)-3-methyl­benzamide

**DOI:** 10.1107/S1600536810011530

**Published:** 2010-03-31

**Authors:** Vinola Z. Rodrigues, Miroslav Tokarčík, B. Thimme Gowda, Jozef Kožíšek

**Affiliations:** a Department of Chemistry, Mangalore University, Mangalagangotri 574 199, Mangalore, India; b Faculty of Chemical and Food Technology, Slovak Technical University, Radlinského 9, SK-812 37 Bratislava, Slovak Republic

## Abstract

In the mol­ecular structure of the title compound, C_16_H_17_NO, the N—H and C=O bonds are *anti* to each other. The two aromatic rings make a dihedral angle of 73.3 (1)°. In the crystal, inter­molecular N—H⋯O hydrogen bonds connect the mol­ecules into *C*(4) chains running along the *c* axis.

## Related literature

For preparation of the title compound and related structures, see: Gowda *et al.* (2008**a* ▶,b* ▶, 2009 ▶); Bowes *et al.* (2003 ▶).
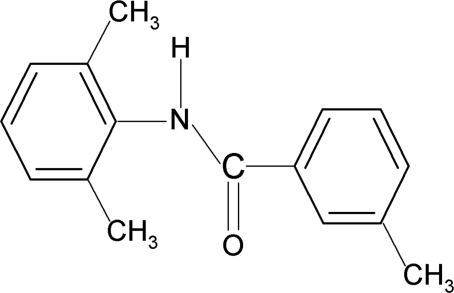


## Experimental

### 

#### Crystal data

C_16_H_17_NO
*M* *_r_* = 239.31
Monoclinic, 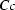

*a* = 12.0715 (4) Å
*b* = 12.4966 (3) Å
*c* = 9.7027 (3) Å
β = 112.123 (4)°
*V* = 1355.92 (7) Å^3^
*Z* = 4
Mo *K*α radiation
μ = 0.07 mm^−1^
*T* = 295 K
0.55 × 0.30 × 0.18 mm

#### Data collection

Oxford Diffraction Xcalibur Ruby Gemini diffractometer
Absorption correction: analytical (*CrysAlis PRO*; Oxford Diffraction, 2009 ▶) *T*_min_ = 0.972, *T*_max_ = 0.989
11288 measured reflections
1371 independent reflections
1260 reflections with *I* > 2σ(*I*)
*R*_int_ = 0.030

#### Refinement

*R*[*F* ^2^ > 2σ(*F* ^2^)] = 0.030
*wR*(*F* ^2^) = 0.084
*S* = 1.08
1371 reflections
169 parameters
3 restraints
H atoms treated by a mixture of independent and constrained refinement
Δρ_max_ = 0.09 e Å^−3^
Δρ_min_ = −0.11 e Å^−3^

### 

Data collection: *CrysAlis PRO* (Oxford Diffraction, 2009 ▶); cell refinement: *CrysAlis PRO*; data reduction: *CrysAlis PRO*; program(s) used to solve structure: *SHELXS97* (Sheldrick, 2008 ▶); program(s) used to refine structure: *SHELXL97* (Sheldrick, 2008 ▶); molecular graphics: *ORTEP-3* (Farrugia, 1997 ▶) and *DIAMOND* (Brandenburg, 2002 ▶); software used to prepare material for publication: *SHELXL97*, *PLATON* (Spek, 2009 ▶) and *WinGX* (Farrugia, 1999 ▶).

## Supplementary Material

Crystal structure: contains datablocks I, global. DOI: 10.1107/S1600536810011530/bt5228sup1.cif

Structure factors: contains datablocks I. DOI: 10.1107/S1600536810011530/bt5228Isup2.hkl

Additional supplementary materials:  crystallographic information; 3D view; checkCIF report

## Figures and Tables

**Table 1:** Hydrogen-bond geometry (Å, °)

*D*—H⋯*A*	*D*—H	H⋯*A*	*D*⋯*A*	*D*—H⋯*A*
N1—H1*N*⋯O1^i^	0.88 (2)	2.09 (2)	2.902 (2)	154 (2)

Symmetry code: (i) 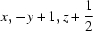.

**Fig. 1.**
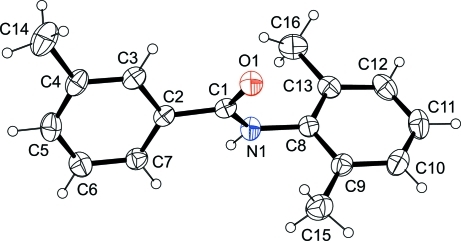
Molecular structure of (I) showing the atom labelling scheme. Displacement ellipsoids are drawn at the 30% probability level and H atoms are represented as small spheres of arbitrary radii.

**Fig. 2.**
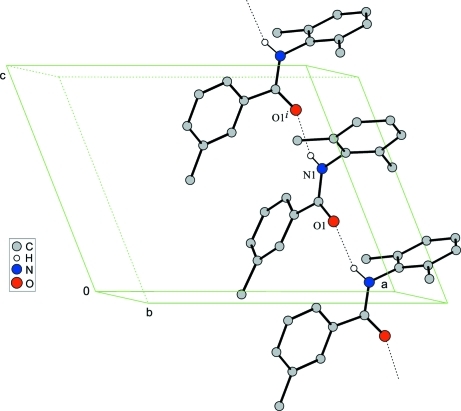
Part of crystal structure of (I) showing molecular chains running along the c-axis. Hydrogen bonds are shown as dashed lines. H atoms not involved in hydrogen bonding were omitted. Symmetry code:(i) x, -y+1, z+1/2.

## supplementary crystallographic information

### Comment

As part of a study of the substituent effects on the crystal structures of benzanilides (Gowda *et al.*, 2008*a,b*, 2009), in the present work, the structure of *N*-(2,6-dimethylphenyl)3-methylbenzamide (I) has been determined. In the structure, the conformations of the N—H and C=O bonds are *anti* to each other (Fig. 1), similar to those observed in *N*-(phenyl)3-methylbenzamide (II)(Gowda *et al.*, 2008*a*), *N*-(2,6-dimethylphenyl)2-methylbenzamide (III) (Gowda *et al.*, 2008*b*), *N*-(2,6-dichloromethylphenyl)- 3-methylbenzamide (IV)(Gowda *et al.*, 2009) and the parent benzanilide (Bowes *et al.*, 2003). Further, the conformation of the C=O bond in (I) is *syn* to the *meta*-methyl substituent in the benzoyl ring, similar to that observed in (III) and (IV), but contrary to the *anti* conformation observed between the C=O bond and the *meta*-methyl group in the benzoyl ring of (II).

The two aromatic rings make a dihedral angle of 73.3 (1) °. The amide group –NH–C(=O)– is twisted by 81.0 (1)° and 25.8 (2)° out of the planes of the 2,6-dimethylphenyl and 3-methylphenyl rings, respectively. In the crystal, intermolecular N–H···O hydrogen bonds (Table 1) connect the molecules into chains running along the c-axis (Fig. 2).

### Experimental

The title compound was prepared according to the literature method (Gowda *et al.*, 2008*a,b*). The purity of the compound was checked by determining its melting point. It was characterized by recording its infrared and NMR spectra. Single crystals of the title compound used in X-ray diffraction studies were obtained from a slow evaporation of its ethanolic solution at room temperature.

### Refinement

H atoms bounded to carbon atoms were positioned with idealized geometry using a riding model with C–H = 0.93 Å or 0.96 Å. The coordinates of the amide H atom were refined with the N–H distance restrained to 0.86 (2) Å. The *U*_iso_(H) values were set at 1.2*U*_eq_(C_aromatic_, N) and 1.5*U*_eq_(C_methyl_). In the absence of significant anomalous scattering, the absolute structure could not be reliably determined and   Friedel pairs were merged. Any references to the Flack parameter were removed.

### Crystal data

**Table:**

C_16_H_17_NO	*F*(000) = 512
*M**_r_* = 239.31	*D*_x_ = 1.172 Mg m^−^^3^
Monoclinic, *C**c*	Mo *K*α radiation, λ = 0.71073 Å
Hall symbol: C -2yc	Cell parameters from 6791 reflections
*a* = 12.0715 (4) Å	θ = 2.3–29.5°
*b* = 12.4966 (3) Å	µ = 0.07 mm^−^^1^
*c* = 9.7027 (3) Å	*T* = 295 K
β = 112.123 (4)°	Block, colourless
*V* = 1355.92 (7) Å^3^	0.55 × 0.30 × 0.18 mm
*Z* = 4	

### Data collection

**Table:**

Oxford Diffraction Xcalibur Ruby Gemini diffractometer	1371 independent reflections
graphite	1260 reflections with *I* > 2σ(*I*)
Detector resolution: 10.434 pixels mm^-1^	*R*_int_ = 0.030
ω scans	θ_max_ = 26.2°, θ_min_ = 2.4°
Absorption correction: analytical (*CrysAlis PRO*; Oxford Diffraction, 2009)	*h* = −14→14
*T*_min_ = 0.972, *T*_max_ = 0.989	*k* = −15→15
11288 measured reflections	*l* = −12→12

### Refinement

**Table:**

Refinement on *F*^2^	Primary atom site location: structure-invariant direct methods
Least-squares matrix: full	Secondary atom site location: difference Fourier map
*R*[*F*^2^ > 2σ(*F*^2^)] = 0.030	Hydrogen site location: inferred from neighbouring sites
*wR*(*F*^2^) = 0.084	H atoms treated by a mixture of independent and constrained refinement
*S* = 1.08	*w* = 1/[σ^2^(*F*_o_^2^) + (0.0513*P*)^2^ + 0.1381*P*] where *P* = (*F*_o_^2^ + 2*F*_c_^2^)/3
1371 reflections	(Δ/σ)_max_ < 0.001
169 parameters	Δρ_max_ = 0.09 e Å^−^^3^
3 restraints	Δρ_min_ = −0.11 e Å^−^^3^

### Special details

**Table:**

Geometry. All esds (except the esd in the dihedral angle between two l.s. planes) are estimated using the full covariance matrix. The cell esds are taken into account individually in the estimation of esds in distances, angles and torsion angles; correlations between esds in cell parameters are only used when they are defined by crystal symmetry. An approximate (isotropic) treatment of cell esds is used for estimating esds involving l.s. planes.
Refinement. Refinement of *F*^2^ against ALL reflections. The weighted *R*-factor wR and goodness of fit *S* are based on *F*^2^, conventional *R*-factors *R* are based on *F*, with *F* set to zero for negative *F*^2^. The threshold expression of *F*^2^ > σ(*F*^2^) is used only for calculating *R*-factors(gt) etc. and is not relevant to the choice of reflections for refinement. *R*-factors based on *F*^2^ are statistically about twice as large as those based on *F*, and *R*- factors based on ALL data will be even larger.

### Fractional atomic coordinates and isotropic or equivalent isotropic displacement parameters (Å^2^)

**Table:**

	*x*	*y*	*z*	*U*_iso_*/*U*_eq_	
C1	0.78435 (17)	0.48438 (15)	0.42193 (19)	0.0400 (4)	
C2	0.67477 (16)	0.55392 (15)	0.3679 (2)	0.0411 (4)	
C3	0.58869 (18)	0.53174 (18)	0.2278 (2)	0.0482 (5)	
H3	0.6004	0.4745	0.1736	0.058*	
C4	0.48627 (19)	0.5928 (2)	0.1676 (2)	0.0558 (5)	
C5	0.4723 (2)	0.6792 (2)	0.2490 (3)	0.0622 (6)	
H5	0.4047	0.7221	0.2095	0.075*	
C6	0.5568 (2)	0.7029 (2)	0.3876 (3)	0.0602 (5)	
H6	0.5457	0.7612	0.4407	0.072*	
C7	0.65776 (19)	0.64009 (17)	0.4474 (2)	0.0486 (5)	
H7	0.7144	0.6556	0.5413	0.058*	
C8	0.95159 (17)	0.41695 (16)	0.63591 (19)	0.0422 (4)	
C9	1.05908 (18)	0.46294 (17)	0.6440 (2)	0.0494 (5)	
C10	1.1631 (2)	0.4041 (2)	0.7137 (3)	0.0621 (6)	
H10	1.236	0.4327	0.7202	0.074*	
C11	1.1606 (2)	0.3050 (2)	0.7730 (3)	0.0677 (7)	
H11	1.2314	0.2674	0.8199	0.081*	
C12	1.0537 (3)	0.2613 (2)	0.7630 (3)	0.0631 (6)	
H12	1.0529	0.1939	0.8032	0.076*	
C13	0.9464 (2)	0.31583 (16)	0.6939 (2)	0.0496 (5)	
C14	0.3930 (3)	0.5643 (3)	0.0162 (3)	0.0890 (9)	
H14A	0.3383	0.6229	−0.0201	0.134*	
H14B	0.35	0.5018	0.0251	0.134*	
H14C	0.4318	0.5502	−0.052	0.134*	
C15	1.0634 (3)	0.5708 (2)	0.5791 (4)	0.0742 (7)	
H15A	1.1447	0.5955	0.6142	0.111*	
H15B	1.0159	0.6203	0.609	0.111*	
H15C	1.0326	0.5658	0.4726	0.111*	
C16	0.8297 (3)	0.2675 (2)	0.6825 (3)	0.0724 (7)	
H16A	0.8367	0.191	0.687	0.109*	
H16B	0.7684	0.2881	0.5898	0.109*	
H16C	0.8091	0.2926	0.7633	0.109*	
N1	0.84288 (14)	0.47696 (14)	0.56895 (16)	0.0447 (4)	
H1N	0.813 (2)	0.5097 (19)	0.627 (3)	0.054*	
O1	0.81747 (13)	0.43660 (12)	0.33310 (16)	0.0518 (4)	

### Atomic displacement parameters (Å^2^)

**Table:**

	*U*^11^	*U*^22^	*U*^33^	*U*^12^	*U*^13^	*U*^23^
C1	0.0423 (9)	0.0448 (9)	0.0324 (9)	−0.0060 (8)	0.0137 (7)	−0.0005 (8)
C2	0.0397 (9)	0.0481 (10)	0.0340 (9)	−0.0046 (8)	0.0120 (7)	0.0050 (8)
C3	0.0482 (11)	0.0587 (12)	0.0359 (10)	−0.0064 (9)	0.0138 (9)	0.0009 (9)
C4	0.0458 (11)	0.0759 (14)	0.0402 (11)	−0.0028 (10)	0.0099 (9)	0.0115 (10)
C5	0.0512 (12)	0.0728 (15)	0.0604 (14)	0.0135 (11)	0.0185 (11)	0.0195 (12)
C6	0.0631 (13)	0.0576 (12)	0.0598 (14)	0.0085 (11)	0.0229 (11)	0.0032 (10)
C7	0.0487 (10)	0.0525 (11)	0.0408 (10)	−0.0012 (9)	0.0125 (8)	−0.0009 (9)
C8	0.0453 (10)	0.0484 (10)	0.0304 (8)	0.0037 (8)	0.0114 (8)	−0.0028 (7)
C9	0.0473 (11)	0.0570 (12)	0.0418 (11)	−0.0009 (9)	0.0146 (9)	−0.0057 (9)
C10	0.0456 (12)	0.0837 (16)	0.0536 (13)	0.0025 (11)	0.0149 (10)	−0.0075 (12)
C11	0.0613 (15)	0.0842 (18)	0.0515 (12)	0.0281 (13)	0.0141 (11)	0.0041 (12)
C12	0.0868 (17)	0.0527 (13)	0.0523 (13)	0.0181 (12)	0.0291 (12)	0.0080 (10)
C13	0.0605 (11)	0.0510 (11)	0.0376 (9)	0.0016 (10)	0.0187 (9)	−0.0024 (8)
C14	0.0626 (16)	0.133 (3)	0.0529 (15)	0.0034 (17)	0.0006 (13)	0.0060 (16)
C15	0.0653 (15)	0.0700 (15)	0.0866 (19)	−0.0104 (13)	0.0280 (14)	0.0054 (14)
C16	0.0841 (17)	0.0659 (15)	0.0730 (16)	−0.0121 (13)	0.0361 (14)	0.0059 (13)
N1	0.0446 (9)	0.0578 (10)	0.0314 (8)	0.0074 (7)	0.0141 (7)	0.0007 (7)
O1	0.0549 (8)	0.0638 (9)	0.0357 (7)	0.0053 (7)	0.0161 (6)	−0.0030 (6)

### Geometric parameters (Å, °)

**Table:**

C1—O1	1.232 (2)	C9—C15	1.496 (3)
C1—N1	1.336 (2)	C10—C11	1.371 (4)
C1—C2	1.502 (3)	C10—H10	0.93
C2—C7	1.385 (3)	C11—C12	1.370 (4)
C2—C3	1.392 (3)	C11—H11	0.93
C3—C4	1.381 (3)	C12—C13	1.392 (3)
C3—H3	0.93	C12—H12	0.93
C4—C5	1.386 (4)	C13—C16	1.498 (4)
C4—C14	1.518 (3)	C14—H14A	0.96
C5—C6	1.380 (3)	C14—H14B	0.96
C5—H5	0.93	C14—H14C	0.96
C6—C7	1.381 (3)	C15—H15A	0.96
C6—H6	0.93	C15—H15B	0.96
C7—H7	0.93	C15—H15C	0.96
C8—C13	1.394 (3)	C16—H16A	0.96
C8—C9	1.394 (3)	C16—H16B	0.96
C8—N1	1.437 (3)	C16—H16C	0.96
C9—C10	1.391 (3)	N1—H1N	0.875 (17)
			
O1—C1—N1	122.20 (18)	C12—C11—C10	119.9 (2)
O1—C1—C2	120.72 (16)	C12—C11—H11	120
N1—C1—C2	117.08 (16)	C10—C11—H11	120
C7—C2—C3	119.08 (17)	C11—C12—C13	121.3 (2)
C7—C2—C1	123.44 (16)	C11—C12—H12	119.3
C3—C2—C1	117.45 (17)	C13—C12—H12	119.3
C4—C3—C2	121.5 (2)	C12—C13—C8	117.6 (2)
C4—C3—H3	119.2	C12—C13—C16	121.1 (2)
C2—C3—H3	119.2	C8—C13—C16	121.3 (2)
C3—C4—C5	118.1 (2)	C4—C14—H14A	109.5
C3—C4—C14	120.0 (2)	C4—C14—H14B	109.5
C5—C4—C14	121.9 (2)	H14A—C14—H14B	109.5
C6—C5—C4	121.3 (2)	C4—C14—H14C	109.5
C6—C5—H5	119.4	H14A—C14—H14C	109.5
C4—C5—H5	119.4	H14B—C14—H14C	109.5
C5—C6—C7	119.9 (2)	C9—C15—H15A	109.5
C5—C6—H6	120	C9—C15—H15B	109.5
C7—C6—H6	120	H15A—C15—H15B	109.5
C6—C7—C2	120.05 (19)	C9—C15—H15C	109.5
C6—C7—H7	120	H15A—C15—H15C	109.5
C2—C7—H7	120	H15B—C15—H15C	109.5
C13—C8—C9	122.22 (18)	C13—C16—H16A	109.5
C13—C8—N1	118.92 (18)	C13—C16—H16B	109.5
C9—C8—N1	118.84 (18)	H16A—C16—H16B	109.5
C10—C9—C8	117.4 (2)	C13—C16—H16C	109.5
C10—C9—C15	120.8 (2)	H16A—C16—H16C	109.5
C8—C9—C15	121.80 (19)	H16B—C16—H16C	109.5
C11—C10—C9	121.6 (2)	C1—N1—C8	123.02 (16)
C11—C10—H10	119.2	C1—N1—H1N	118.3 (17)
C9—C10—H10	119.2	C8—N1—H1N	118.7 (17)
			
O1—C1—C2—C7	−153.25 (19)	C13—C8—C9—C15	179.0 (2)
N1—C1—C2—C7	26.9 (3)	N1—C8—C9—C15	−2.6 (3)
O1—C1—C2—C3	24.4 (3)	C8—C9—C10—C11	−0.3 (3)
N1—C1—C2—C3	−155.44 (17)	C15—C9—C10—C11	−179.7 (2)
C7—C2—C3—C4	−0.8 (3)	C9—C10—C11—C12	0.6 (4)
C1—C2—C3—C4	−178.60 (18)	C10—C11—C12—C13	−0.2 (3)
C2—C3—C4—C5	1.6 (3)	C11—C12—C13—C8	−0.4 (3)
C2—C3—C4—C14	−178.4 (2)	C11—C12—C13—C16	179.6 (2)
C3—C4—C5—C6	−1.2 (3)	C9—C8—C13—C12	0.7 (3)
C14—C4—C5—C6	178.8 (3)	N1—C8—C13—C12	−177.66 (18)
C4—C5—C6—C7	0.1 (3)	C9—C8—C13—C16	−179.3 (2)
C5—C6—C7—C2	0.6 (3)	N1—C8—C13—C16	2.3 (3)
C3—C2—C7—C6	−0.3 (3)	O1—C1—N1—C8	2.8 (3)
C1—C2—C7—C6	177.36 (19)	C2—C1—N1—C8	−177.37 (17)
C13—C8—C9—C10	−0.4 (3)	C13—C8—N1—C1	−101.4 (2)
N1—C8—C9—C10	177.99 (18)	C9—C8—N1—C1	80.2 (2)

### Hydrogen-bond geometry (Å, °)

**Table:**

*D*—H···*A*	*D*—H	H···*A*	*D*···*A*	*D*—H···*A*
N1—H1N···O1^i^	0.88 (2)	2.09 (2)	2.902 (2)	154 (2)

Symmetry codes: (i) *x*, −*y*+1, *z*+1/2.
